# Unconscious avoidance of eye contact in autism spectrum disorder

**DOI:** 10.1038/s41598-017-13945-5

**Published:** 2017-10-17

**Authors:** Apoorva Rajiv Madipakkam, Marcus Rothkirch, Isabel Dziobek, Philipp Sterzer

**Affiliations:** 1 Visual Perception Laboratory, Department of Psychiatry, Charité – Universitätsmedizin Berlin, corporate member of Freie Universität Berlin, Humboldt-Universität zu Berlin, and Berlin Institute of Health, Charitéplatz 1, 10117 Berlin, Germany; 2 International Graduate Program Medical Neurosciences, Charité – Universitätsmedizin Berlin, corporate member of Freie Universität Berlin, Humboldt-Universität zu Berlin, and Berlin Institute of Health, Luisenstr. 56, 10117 Berlin, Germany; 3 Humboldt Universität zu Berlin, Unter den Linden 6, 10099 Berlin, Germany

## Abstract

Atypical responses to direct gaze are one of the most characteristic hallmarks of autism spectrum disorder (ASD). The cause and mechanism underlying this phenomenon, however, have remained unknown. Here we investigated whether the atypical responses to eye gaze in autism spectrum disorder is dependent on the conscious perception of others’ faces. Face stimuli with direct and averted gaze were rendered invisible by interocular suppression and eye movements were recorded from participants with ASD and an age and sex matched control group. Despite complete unawareness of the stimuli, the two groups differed significantly in their eye movements to the face stimuli. In contrast to the significant positive saccadic index observed in the TD group, indicating an unconscious preference to the face with direct gaze, the ASD group had no such preference towards direct gaze and instead showed a tendency to prefer the face with averted gaze, suggesting an unconscious avoidance of eye contact. These results provide the first evidence that the atypical response to eye contact in ASD is an unconscious and involuntary response. They provide a better understanding of the mechanism of gaze avoidance in autism and might lead to new diagnostic and therapeutic interventions.

## Introduction

While verbal communication is the predominant means to deliver messages in social contexts, nonverbal signals play an essential role in paving the way for social interactions. The eyes in particular deliver a multitude of social cues. Adults^1^ and already neonates^2^ shift their attention preferably and reflexively towards others’ direct gaze to establish mutual eye contact. This preference for direct gaze is not limited to conscious perception, but occurs even when individuals are completely unaware of being looked at^3^, accentuating the rapid and automatic processing of direct gaze in typically developed (TD) individuals. In contrast, individuals with autism spectrum disorder (ASD) show atypical responses to eye gaze – in particular an avoidance of mutual eye contact^4^. This striking and well-documented feature of ASD is directly related to deficits in social interactions and occurs early in the course of the disorder^5^. The cause and the mechanisms underlying eye contact avoidance in autism, however, have remained elusive^6^. On the one hand, it could be the consequence of a fast, yet conscious evaluation process involving high-level cortical processing stages, that is, an active avoidance of the eye region^7^. On the other hand and what remains unknown, is whether this avoidance of eye contact could proceed automatically and even unconsciously. Such an unconscious avoidance of eye contact would imply a mechanism implemented at low levels of the processing hierarchy and differences in the neurodevelopmental pathway of gaze processing^6^. A deeper understanding of the degree of the automaticity of gaze avoidance in autism is not only relevant for a refinement of the psychopathological model of autism but could also aid the development of effective treatments.

To determine whether the atypical response to direct gaze in autism is dependent on the awareness of others’ faces, we recorded eye movements from 14 participants with ASD and 20 TD individuals that were matched in age and sex to the patients group while they searched for faces with different gaze directions that were not consciously perceived. Participants’ awareness of the faces was assessed based on trial-by-trial method using both subjective and objective measures. A preference to one of the two face stimuli was quantified with a ‘saccadic preference index’ that was based on the number of eye movements made to a particular stimulus, such that positive values indicated a saccadic preference for direct gaze and negative values for averted gaze^2,3^.

## Results

### Participant characteristics

The ASD participants did not differ from the TD participants in their verbal intelligence (TD: 109.5 ± 1.7 SEM and ASD: 113.3 ± 2.3 SEM; *t*(32) = −1.36, *p* = 0.18). In addition, there were no differences in attention, which was measured by the d2 test (TD: 103.5 ± 2.7 SEM and ASD: 100.2 ± 3.0 SEM; *t*(32) = 0.79, *p* = 0.43). Thus, it is unlikely that attention differences accounted for differences in eye movements during the task. However, with respect to scores on the autism spectrum quotient (AQ) questionnaire, all ASD participants were above the cut-off of 32 with a mean AQ score of 42.42 ± 1.0 SEM, while the TD participants had a mean AQ score of 15.05 ± 1.0 SEM.

### Behavioral data

**Figure 1:**
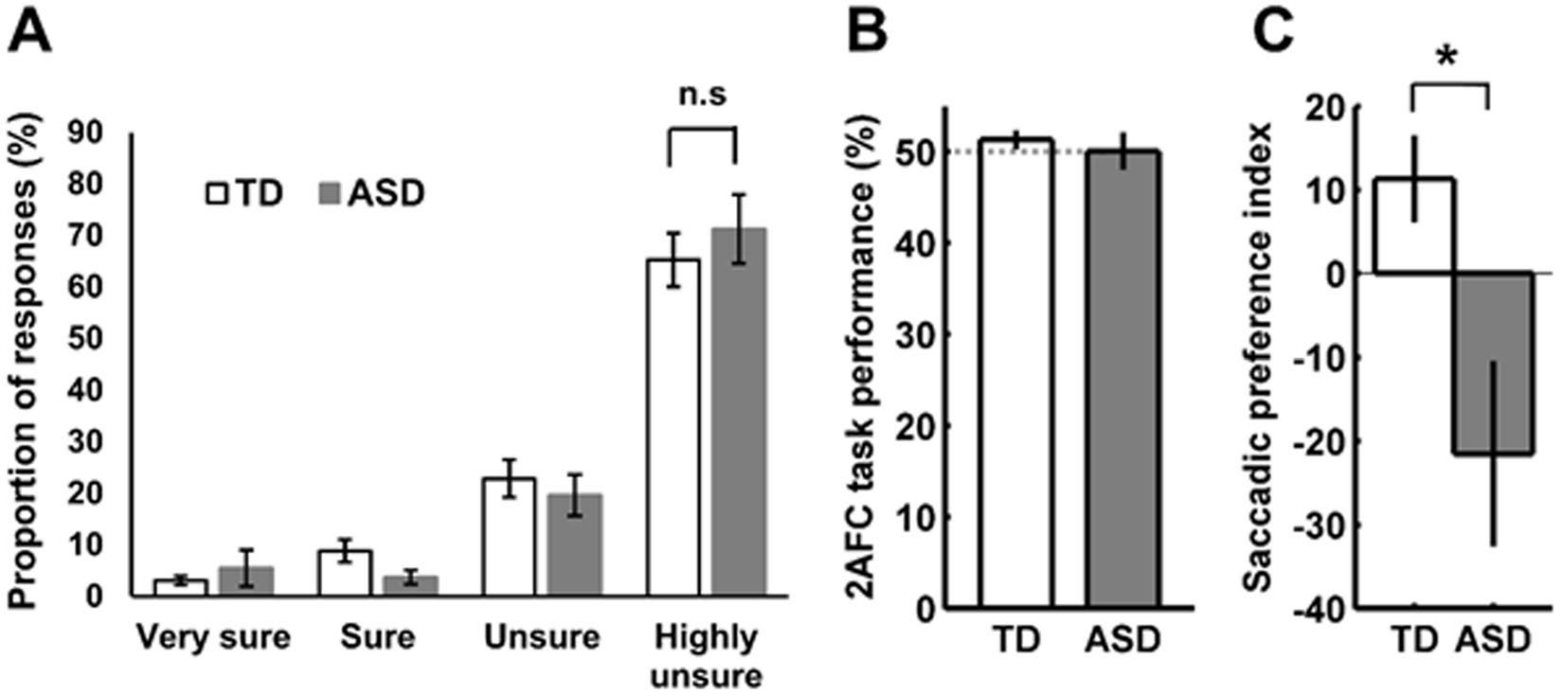
(**A**) Mean proportions of subjective confidence ratings. There was no difference in the number of subjectively unaware (‘highly unsure’) trials, that is, the number of trials used in the analyses between the two groups (t(32) = −0.72, p = 0.48). **(B)** The 2AFC task performances neither statistically differed between the two groups nor were they significantly different from chance level of 50% (dotted line), further indicating participants’ unawareness of the stimuli. **(C)** Statistically significant difference between the saccadic preference indices for the TD and ASD groups (t(18.83) = 2.68, p = 0.015). While the TD group showed a saccadic significant preference to the face with direct gaze, a negative preference index in the ASD group indicated an unconscious avoidance of the face with direct gaze. Error bars indicate within-subject SEM.

Participants’ subjective awareness was assessed trial wise using a four-point confident rating scale that ranged from very sure to very unsure. In addition, their task performance in a 2-alternative forced choice (2-AFC) task was used to assess their awareness on objective criteria. The two groups did not differ in how often they indicated to be subjectively unaware of the stimuli (TD: 65.2% ± 5.2 SEM and ASD: 71.2% ± 6.8 SEM; *t*(32) = −0.71, *p* = 0.48) (Fig. 1A). In these least confident trials, the average 2AFC performances neither significantly differed between groups (*t*(32) = 0.61; *p* = 0.55) nor from chance level (50%) within each group (TD: *t*(19) = 1.32, *p* = 0.20; ASD: *t*(13) = 0.04, *p* = 0.97; one sample t-tests) (Fig. 1B). In addition, a Bayesian analysis for uniform distributions above and below 50% revealed Bayes factors that smaller than 0.33 for both groups (TD: 0.11 and 0.01; ASD: 0.05 and 0.05), providing substantial evidence for the null hypothesis, that is, no difference from chance level.

### Eye tracking data

Despite complete unawareness of the stimuli, the two groups differed significantly in their eye movements towards the face stimuli (*t*(18.83) = 2.68, *p* = 0.015; degrees of freedom are adjusted for unequal variances by the Welch-Satterthwaite method) (Fig. 1C). Critically, while the TD group had a strong unconscious preference for the faces with direct gaze^3^ (M = 11.36% ± 5.2 SEM; *t*(19) = 2.16, *p* = 0.043; one sample t-test against 0), participants in the ASD group not only lacked such a preference for direct gaze but instead tended to show a priority for averted gaze (M = −21.52% ± 11.1 SEM; t(13) = −1.94, p = 0.07), suggesting an unconscious avoidance of eye contact in this group. Overall, the proportion of trials in which a saccade was made was similar in the TD and ASD groups (TD: 71.8% ± 4.5 SEM; ASD: 68.3% ± 5.9 SEM; *t*(32) = 0.47, *p* = 0.64), indicating that the difference in saccadic preference was not due to a systematic group difference in the frequency of eye movements.

## Discussion

In this study we replicate our previous finding that TD individuals have an unconscious bias towards faces with direct gaze^3^ while showing that adults with ASD do not show such a bias, but, to the contrary, a tendency towards preferring faces with averted gaze. This finding provides the first evidence that the atypical responses to eye contact commonly observed in ASD do not depend on the conscious perception of others’ faces and their gaze directions.

Previous research on the extent to which atypical responses to others’ eye gaze in autism are based on automatic processes^6^ have produced inconsistent results. In response to visible face stimuli, patients with ASD show fast and reflexive-like eye movements away from the eye region^7^, while in other tasks they do not exhibit impairments in rapidly orienting towards others’ eye gaze^8^. However, responses to visible face stimuli could, at least in part, be based on voluntary or strategic processes. In contrast, behavioural responses to stimuli that are suppressed from awareness preclude the influence of voluntary and strategic processes since in this case, the individual completely lacks any conscious knowledge about the stimulus. Previous research also showed that the access to awareness, that is, the detection of an initially suppressed stimulus, is faster for faces with direct versus averted gaze in TD but not in ASD^9^. However, it is critical to note that this finding does not allow for the conclusion that atypical responses to eye contact in ASD are independent of visual awareness, as this approach does not provide unequivocal evidence for the processing of stimuli in the absence of awareness^10–12^.

The current results therefore go substantially beyond these previous findings by showing that, even when face stimuli are completely suppressed from awareness (as evidenced by a meticulous trial-by-trial assessment of awareness using subjective and objective measures), individuals with ASD avoid eye contact. This suggests that atypical responses to eye gaze in ASD do not require the recruitment of high-level brain processes for the active avoidance of eye-contact; rather, these responses may rely on lower-level brain circuits that are thought to underlie fast and automatic eye gaze processing, involving subcortical structures via magnocellular channels^4,13^. The magnocellular pathway is crucial for transmitting low spatial frequency information to the amygdala, the successful functioning of which is required for the processing of social and salient information^14,15^. Indeed, the amygdala is directly involved in detecting salient facial features and drives reflexive eye movements towards them^16^. It has further been previously suggested that altered visual scan paths for faces in ASD are related to a hypofunction of the amygdala^17^.

During typical development, early sensitivity to eye gaze develops rapidly through the magnocellular system^2,6,18^. In ASD, neurodevelopmental abnormalities in magnocellular cells are thought to lead to disruptions in brain networks involved in social orienting, which could result in a decreased saliency and sensitivity to eye gaze information^4^. Our finding of unconscious eye contact avoidance could thus be the direct consequence of such a magnocellular processing deficit. Alternatively, or in addition, it is conceivable that individuals with ASD consciously avoid eye contact throughout life (e.g., to reduce negative arousal^19^), causing an impaired sensitivity to direct gaze due to reduced exposure, which may in turn contribute to altered unconscious processing of gaze cues in the long run. Together, both of the above theories could lead to the reported abnormal amygdala responses to eye gaze in ASD^20,21^.

Whether the unconscious avoidance of eye contact reflects a basic magnocellular deficit in gaze processing or rather a consequence of the voluntary avoidance of eye contact throughout development is an intriguing question that will require the investigation of unconscious gaze processing early in the course of the disorder. Akin to atypical brain function^22,23^, we speculate that unconscious biases may even be present in ASD before overt behavioral phenotypes. Supporting the interpretation that an early basic deficit in eye gaze processing underlies unconscious eye contact avoidance, a recent study reported that in children with autism reduced attention to the eyes is due to a passive insensitivity to social signals^24^.

It has been recently proposed that the employment of eye tracking may prove beneficial for the screening of and differentiation between patients with ASD^25^. Eye tracking and gaze-contingent designs in particular provide further insights into face processing^26^ as well as a better understanding of social interactions in ASD^27^. In light of evidence showing that neural responses to eye gaze in infants predict the later development of autism^24^, our present results offer a promising starting point for investigating the extent to which altered automatic eye gaze processing is predictive of the later development of autism. Unconscious avoidance of eye contact may provide a useful marker for participants’ automatic attentional preferences unbiased by voluntary responses.

Finally, a potentially important therapeutic consequence of our current results could be that interventions, which currently focus on only overt social deficits^28,29^, shift their focus to include the training of fast and reflexive reactions, for example by reinforcing reflexive shifts to direct gaze with a reward. Such a strategy could help overcome obstacles in the treatment of impaired social functioning that are due to an active avoidance of social signals.

To our knowledge, this is the first study providing direct evidence for the unconscious avoidance of eye contact in individuals with ASD. By showing that atypical responses to eye gaze in ASD can occur unconsciously, and by thus indicating a highly automatic avoidance of eye contact, our findings go substantially beyond the current literature on gaze processing and provide a deeper understanding of the mechanisms underlying impaired social functioning in ASD. Finally, our current results pave the way for future investigations into unconscious gaze processing in infants and provide a starting point for the development of new therapeutic interventions targeting impaired sensitivity to social cues in autistic individuals.

## Methods

### Participants

Seventeen adults with ASD and twenty-two TD controls participated in the study. Three participants with ASD and two from the TD group had to be excluded from all analyses due to difficulties while acquiring the eye tracking data (excessive head movements, blinks, etc.), resulting in poor data quality. The final sample consisted of 14 adults with ASD (8 males; mean age: 35.4 ± 2.3 (SEM) years) and 20 TD adults (10 males; mean age: 35.3 ± 1.8 (SEM) years. Both groups were matched for chronological age and gender. All participants performed a test for verbal intelligence (Mehrfachwahl-Wortschatz-Test (WST)^30^ and the d2 test of attention^31^. Scores from the verbal intelligence test corresponded to participants’ first attempt of the test. ASD diagnoses were confirmed by clinical experts from specialized clinics according to the ICD-10 criteria for Asperger syndrome and autism without intellectual difficulties. In addition, all participants filled out the autism spectrum quotient (AQ) questionnaire which has shown to be a reliable scale for the measure of autistic traits in adults of normal intelligence^32^. The AQ is a self-report questionnaire with scores ranging from 0 to 50. Higher scores indicate more autistic traits and scores above 32 indicate clinical levels of autism^32^. Furthermore, for 11 of the 14 participants, diagnosis was substantiated by the Autism Diagnostic Observation Schedule (ADOS-G; mean: 11.4 ± 1.1 (SEM), cut-off autism spectrum: 7) (Lord *et al*., 1994). All participants were invited to take part in the study if they had not been taking any psychotropic medication in the last six months. Further, the Structured Clinical Interview for Axis I Disorders (SCID-I) was carried out to control for comorbidities in the ASD group and to rule out psychiatric disorders in the control group. ASD adults were recruited through an online forum of an ASD advocate group (Aspies e.V.) and through the outpatient clinic of the Charité – Universitätsmedizin Berlin. The control group was recruited by local advertisement. All participants had normal or corrected-to-normal vision, received payment for their participation and written informed consent was obtained prior to the start of the study. The study was conducted in accordance with the 2008 World Medical Association Declaration of Helsinki and was approved by the local ethics committee of the Charité-Universitätsmedizin Berlin.

### Stimuli

Three greyscale, female face exemplars that have been used in a number of previous studies investigating gaze directions^1,3,33^ were the main stimuli in the current experiment. There were two versions of each face exemplar: one with direct and the other with an averted gaze. The faces had laterally averted heads and only the irises were shifted within the eyes for the two gaze directions, avoiding low-level stimulus confounds between the stimuli. The faces were cut to oval shapes comprising a size of 3.8° × 4.5° (width × height) and were equalized for global contrast (root mean square contrast of 0.05) and luminance. All visual stimuli were presented with Matlab (The MathWorks, Natick, MA, USA), using the Cogent 2000 toolbox (www.vislab.ucl.ak.uk/cogent.php) on a 19-inch CRT monitor (resolution: 1024 × 768 Px; refresh rate: 60 Hz). Participants viewed the screen at a distance of 60 cm through a mirror stereoscope, which provided separate input to the two eyes. Participants’ heads were stabilised by a chin rest and their eye movements were recorded with a high-speed video-based eyetracker (Cambridge Research Systems, UK; sampling rate: 250 Hz; spatial accuracy: 0.05°).

## Procedure

The experimental design was similar to the study by Rothkirch *et al*.^3^. First, participants’ dominant eye was determined using an eye dominance test^34^. Details of the eye dominance test are explained in a previous publication^3^. Both, the eye dominance test and the main experiment used the method of continuous flash suppression to render the stimuli invisible to participants^35^. In this method, a low-contrast, static stimulus of interest, which is presented to one eye, is suppressed from awareness by the simultaneous presentation of high-contrast, dynamic mask images to the other eye.

**Figure 2:**
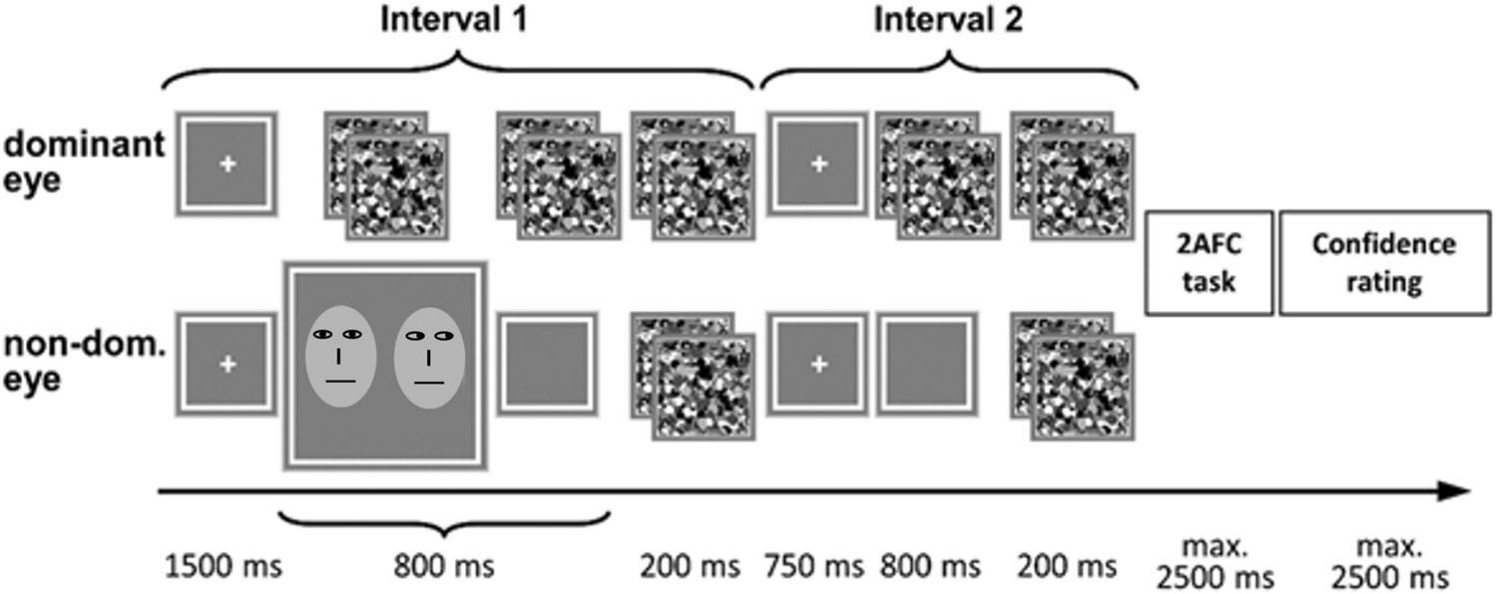
Trial structure. Continuous flash suppression was used to suppress the stimuli from awareness. Participants made eye movements to search for the face stimuli and indicated in a 2-alternative forced choice (2AFC) task the interval in which the stimuli were presented. Trials ended with a confidence rating scale, which is a measure of participants’ subjective awareness of the stimuli. Note: Schematic faces with direct and averted gaze are only used for depiction purposes. The actual stimuli used in the experiment had laterally averted heads (see section Stimuli) and can be found in a previous publication^3^.

In the main experiment, all stimuli were presented within a white frame (12° × 12°) with a grey background (luminance: 30 cd/m²). Trials started with a white central fixation cross (0.6° × 0.6°) for 1500 ms (Fig. 2). If participants did not fixate the cross, the cross was thickened and displayed on the screen till central fixation was established. Then, two intervals each with a duration of 800 ms followed, during which high-contrast greyscale mask stimuli (12° × 12°) were flashed to the participant’s dominant eye at a frequency of 10 Hz to induce continuous flash suppression. Two low-contrast face stimuli (root mean square contrast of 0.03), one with direct and the other with an averted gaze were presented concurrently with the masks. The face stimuli were presented to the non-dominant eye, in one of the two intervals, within the left and right half of the white frame (eccentricity: 3.4°). Both face stimuli were presented either 3° above, below, or at the horizontal meridian. Participants’ task was to actively search for the faces by making eye movements. Upon the first eye movement that landed on a face, both faces were removed from the screen which helped reduce the risk of participants becoming aware of the faces^3^. The two intervals were separated by a fixation period of 750 ms and each interval was followed by the presentation of CFS masks for 200 ms to both eyes, to prevent after images. After the second interval, participants were prompted with two questions. The first was a two-alternative forced-choice (2AFC) task where they indicated the interval in which the face stimuli were presented. In the second question, participants had to indicate their confidence in their 2AFC decision on a four-point confidence rating scale, ranging from ‘very sure’ to ‘very unsure’. The confidence rating scale provides a subjective measure of participants’ awareness of the face stimuli^36^.

The experiment consisted of 8 runs of 38 trials each. Each run had two ‘dummy’ trials where the face stimuli were presented at full contrast to the dominant eye. These trials were included to maintain participants’ motivation to search for the faces and were discarded from all analyses. Prior to the start of each run, a nine-point calibration of the eye tracker was performed. All conditions, i.e. the allocation to one of the two intervals and the spatial location of the faces was randomized and counter-balanced across trials.

### Data Analyses

#### Analyses of behavioral data

For each participant, trials in which they indicated to be least confident were selected and included in all further analyses. To test whether participants were able to discriminate the face-present from the face-absent intervals despite their low confidence, a one sample t-test was performed on the 2AFC task performances of both groups against the chance level of 50%. However, since a non-significant result of a t-test does not provide conclusive evidence for the null hypothesis^37^, we additionally performed a Bayesian analysis using an online Bayes calculator (http://www.lifesci.sussex.ac.uk/home/Zoltan_Dienes/inference/bayes_factor.swf). The Bayes analysis was performed on a uniform distribution varying from 0 to 50% and from 50 to 100% to test that the data were neither significantly above or below chance. Bayes factors (BF10) < 0.33 provide substantial evidence for the null over the alternative hypothesis, BF10 > 3 can be interpreted as evidence for the alternative over the null hypothesis^38^.

#### Analyses of eye tracking data

Pre-processing of eye tracking data comprised interpolation of missing data points on the basis of a cubic-spline interpolation if no more than 24 ms of consecutive data were missing. Then low-pass filtering using a second order Savitzky-Golay filter was performed. Trials were included in the analyses if they fulfilled the following criteria: (i) participants’ made a manual response to both questions: the 2AFC task and the confidence rating, (ii) participants indicated the lowest level of confidence, (iii) after pre-processing at least 95% of the eyetracking data collected during a trial were available and not lost due to blinks or artefacts.

For the included trials, we calculated the number of first saccades made towards one of the two face stimuli. A saccade was defined as an eye movement that exceeded a velocity of 60°/s for at least 12 ms. Eye movements starting earlier than 100 ms after stimulus onset, were excluded from the analysis. A preference to one of the two face stimuli was quantified with a ‘saccadic preference index’, defined by the ratio of the difference between the number of saccades to the direct and averted face stimulus, to their sum. Mathematically, this was 100*(sd − *sa)/(sd* + *sa)*, where sd and sa refer to the number of initial saccades that landed on the face with direct and averted gaze respectively. Thus, positive values indicated a saccadic preference for direct gaze and negative values for averted gaze^2,3^.

### Data Availability

The datasets generated and analysed during the current study are available from the corresponding author on reasonable request.
